# Exploring the oncogenic role and prognostic value of *CKS1B* in human lung adenocarcinoma and squamous cell carcinoma

**DOI:** 10.3389/fgene.2025.1449466

**Published:** 2025-03-17

**Authors:** Md. Solayman Hossain, Tariqul Islam Tusar, Nairita Ahsan Faruqui, Tanjim Ishraq Rahaman, Yasin Arafath Sharker, Shimran Saharia Santo, Abu Tayab Moin, Yusha Araf, Ibrahim Khalil Afif, Shoaib Saikat, Mohammad Jakir Hosen

**Affiliations:** ^1^ Department of Biochemistry and Molecular Biology, Faculty of Biological Sciences, Jahangirnagar University, Dhaka, Bangladesh; ^2^ Department of Pharmacy, University of Asia Pacific, Dhaka, Bangladesh; ^3^ Biotechnology Program, Department of Mathematics and Natural Sciences, School of Data and Sciences, BRAC University, Dhaka, Bangladesh; ^4^ Department of Biotechnology and Genetic Engineering, Faculty of Life Sciences, Bangabandhu Sheikh Mujibur Rahman Science and Technology University, Gopalganj, Bangladesh; ^5^ Department of Genetic Engineering and Biotechnology, Faculty of Biological Sciences, University of Chittagong, Chattogram, Bangladesh; ^6^ Department of Genetic Engineering and Biotechnology, School of Life Sciences, Shahjalal University of Science and Technology, Sylhet, Bangladesh; ^7^ Department of Biotechnology, Bangladesh Agricultural University, Dhaka, Bangladesh; ^8^ Department of Biochemistry and Biotechnology, Faculty of Bio-Sciences, University of Barishal, Barishal, Bangladesh

**Keywords:** *CKS1B* gene, lung adenocarcinoma, lung squamous cell carcinoma, gene expression, biomarker, mRNA expression and regulation, gene mutation, signaling pathways

## Abstract

**Introduction:**

Lung cancer (LC) is a highly aggressive malignancy and remains a leading cause of cancer-related mortality worldwide. Non-small cell lung cancer (NSCLC), which includes adenocarcinoma (LUAD) and squamous cell carcinoma (LUSC), accounts for the majority of these deaths. Due to the lack of early clinical symptoms and late-stage diagnosis, there is an urgent need for precise and targeted therapeutic strategies. Cyclin-dependent kinase regulatory subunit 1B (*CKS1B*), a key regulator of the cell cycle, has been implicated in various human cancers. Emerging evidence suggests that its upregulation is associated with poor prognosis in NSCLC, highlighting its potential as a biomarker for early detection and targeted therapy.

**Methods:**

In this study, we conducted a comprehensive bioinformatics analysis to evaluate the role of *CKS1B* in LUAD and LUSC. Differential gene expression analysis, survival analysis, immune infiltration correlation, and pathway enrichment analysis were performed using publicly available transcriptomic datasets. Additionally, gene interaction networks were analyzed to assess the functional significance of *CKS1B* in lung cancer progression.

**Results:**

Our findings indicate a significant overexpression of *CKS1B* in LUAD and LUSC compared to normal lung tissues. Survival analysis demonstrated that higher *CKS1B* expression correlates with poor prognosis in NSCLC patients. Immune infiltration analysis revealed a potential role of *CKS1B* in modulating the tumor microenvironment, further supporting its relevance in lung cancer progression. Functional enrichment analysis highlighted its involvement in critical oncogenic pathways, including cell cycle regulation and immune modulation.

**Discussion:**

The results suggest that *CKS1B* serves as a potential biomarker for early detection and prognosis in NSCLC. Its association with immune response pathways underscores its possible role in immunotherapy. However, despite these promising findings, further in vivo and in vitro studies are necessary to validate *CKS1B*'s clinical applicability as a diagnostic and therapeutic target for lung cancer.

## 1 Introduction

Cancer represents a complex group of diseases characterized by the uncontrolled proliferation of abnormal cells, often followed by their spread to surrounding tissues (What Is Cancer, 2021). In 2020, the global cancer burden was estimated at 19.3 million new cases and nearly 10 million deaths (Sung et al., 2021). Lung cancer (LC) remains a leading cause of cancer-related mortality, accounting for approximately 18.7% of cancer deaths and 12.4% of new cases annually. Non-small cell lung cancer (NSCLC) dominates LC cases, comprising about 80%–85%, with its two major histologic subtypes, lung adenocarcinoma (LUAD) and lung squamous cell carcinoma (LUSC), representing 40% and 30% of new LC cases, respectively (What Is Lung Cancer, 2021; Bender, 2014).

One of the significant challenges in treating NSCLC lies in its detection at advanced stages (National Lung Screening Trial Research Team, 2019). Early-stage patients often present no clear clinical symptoms, and the lack of effective early detection methods exacerbates the problem (Foss et al., 2011). Recent data highlights this issue, with a notable increase in Stage IV NSCLC diagnoses from 43.3% in 2013 to 49.3% in 2023 (Herms et al., 2024). Furthermore, the overall 5-year survival rate for LC remains at 20%, significantly lower than for most other cancers (Wong et al., 2017; Allemani et al., 2018; Bray et al., 2024). While advancements in therapies have improved outcomes for some, the 5-year survival rate for advanced NSCLC is still less than 15%, while early-stage NSCLC patients experience survival rates as high as 80% (Jemal et al., 2011). These figures emphasize the crucial need for early detection and accurate prognostic markers to improve survival outcomes.

In recent years, an increasing focus has been placed on identifying differentially expressed genes (DEGs) as potential biomarkers for early detection and prognosis of NSCLC subtypes such as LUAD and LUSC (Yu and Tian, 2020a). Tumor development involves a cascade of events including uncontrolled cell growth, genome instability, invasion of nearby tissues, and metastasis, which ultimately result in limited treatment options and poorer outcomes for patients (Shi et al., 2020a). The identification of reliable biomarkers can help in developing targeted therapies that selectively kill tumor cells while sparing healthy tissues, offering hope for improved treatment strategies.

Cyclin-dependent kinase regulatory subunit 1B (*CKS1B*) has emerged as a key player in cell cycle regulation and cancer progression. A member of the conserved cyclin kinase subunit 1 (*CKS1*) protein family, *CKS1B* regulates the eukaryotic mitotic cycle and plays a vital role in normal cell division and growth (Krishnan et al., 2009). Its protein, encoded by the *CKS1B* gene on chromosome 1q21, has a molecular weight of 9 kDa and consists of 79 amino acids. Dysregulation of *CKS1B* is associated with abnormal cell cycle progression, which can contribute to the onset and progression of various malignancies. The *CKS1B* protein interacts with cyclin-dependent kinases (CDKs) and their inhibitors, influencing key processes such as cell proliferation, invasion, metastasis, and chemoresistance (Krishnan et al., 2009; Stella et al., 2014; Zeng et al., 2019; Lee et al., 2011; Zhan et al., 2007).

*CKS1B* overexpression has been documented in several cancers, including hepatocellular carcinoma (Lee et al., 2011), colon cancer (Wang et al., 2010), lung cancer (Zolota et al., 2010), oral squamous cell carcinoma (Kitajima et al., 2004), breast cancer (Slotky et al., 2005), and retinoblastoma (Zeng et al., 2019). Additionally, its role as one of the 70 high-risk genes associated with poor prognosis in multiple myeloma (MM) has been established (Shaughnessy et al., 2006; Chen et al., 2012). These findings suggest that *CKS1B* could serve as a valuable therapeutic target across various malignancies (Huang et al., 2015; Hwang et al., 2019).

Despite the widespread occurrence of LUAD and LUSC, limited biomarkers exist for early detection, and current treatment options often result in suboptimal survival rates. Prognostic markers can help guide treatment strategies by identifying key tumor characteristics, but many biomarkers still lack the necessary sensitivity and specificity for routine clinical use in lung cancer detection (Lemjabbar-Alaoui et al., 2015). Given the crucial role of *CKS1B* in cell cycle regulation and its association with poor prognosis in several cancers, we hypothesized that *CKS1B* expression may serve as a prognostic biomarker for the early diagnosis and treatment of LUAD and LUSC. In this study, we explored the oncogenic role of *CKS1B* in NSCLC subtypes through a bioinformatics approach. Our findings revealed a significant correlation between elevated *CKS1B* expression and poor survival outcomes in LUAD and LUSC, suggesting its potential as a valuable biomarker for early detection and prognosis. The study also highlights the possibility of targeting *CKS1B* for novel therapeutic strategies aimed at improving patient outcomes. Further *in vivo* and *in vitro* investigations are warranted to validate its clinical utility and explore its role in the regulation of key signaling pathways involved in lung cancer progression.

## 2 Methods

### 2.1 Determination of *CKS1B* mRNA expression in cancerous and normal tissues

Three different databases are entitled Oncomine (https://www.oncomine.org) (Rhodes et al., 2004), GEPIA2 (http://gepia2.cancer-pku.cn) (Tang et al., 2019), and GENT2 (http://gent2.appex.kr) (Park et al., 2019) were used to analyze the expression of *CKS1B* expression in multiple cancerous tissues and their normal, non-cancerous counterparts. The Oncomine database contains 715 independent datasets that store experimental information of 86,733 cancer samples and 12,764 standardized tissue samples. This server is well-known for its high reliability, accuracy, consistency, and scalability (Rhodes et al., 2004). This platform was employed to assess gene expression data from curated datasets. In the Oncomine database, the default parameters i.e., p-value threshold of 1E-4, fold change threshold of 2.0, and Top 10% Gene rank, were set. GEPIA2 (Gene Expression Profiling Interactive Analysis) database is widely utilized to analyze the mRNA expression profiling of a variety of genes. The server houses the information of 9,736 cancerous and 8,587 normal tissue samples, extracted from the TCGA (The Cancer Genome Atlas) and GTEx (Genotype-Tissue Expression) projects (Tang et al., 2019). This server integrates TGCA and GTEx data for differential expression and survival analysis. During analysis by the GEPIA2 server, all the parameters were kept at their default values. Along with the above-mentioned servers, GENT2 (Gene Expression database of Normal and Tumor tissues-2) was also used to analyze the expression of the *CKS1B* gene in more than 68,000 tumor and normal tissue samples stored in its database. This server generates reliable and quite accurate outcomes by exploiting the Apache Lucene indexing and Google Web Toolkit (GWT) framework (Park et al., 2019). It is used to analyze gene expression profiles. Finally, the expression of the *CKS1B* gene was observed for both LUAD and LUSC, according to the TIMER analysis (http://timer.cistrome.org/).

### 2.2 Expression profiling of *CKS1B* in cancerous and normal lung tissues

Four different web servers, namely, Oncomine (Rhodes et al., 2004), GEPIA2 (Tang et al., 2019), UALCAN (http://ualcan.path.uab.edu/) (Chandrashekar et al., 2017), and HPA (https://www.proteinatlas.org/) (Uhlén et al., 2015) were used to determine the expression profile of *CKS1B* mRNA in normal and cancerous LUAD and LUSC tissues. UALCAN web-server allows the users to detect novel cancer biomarkers and carry out numerous *in silico* analyses to map the expression of target genes by providing free access to the cancer OMICS datasets like the TCGA database (Chandrashekar et al., 2017). The relative expression pattern of *CKS1B* mRNA in both LUAD and LUSC samples was also analyzed using the TCGA database. The HPA (Human Protein Atlas) database was used to make a visual comparison between LUAD and LUSC tissues and normal lung tissues through immunohistochemistry. This project aims to map all human proteins in cells, tissues, and organs by combining various omics technologies such as mass spectrometry-based proteomics, transcriptomics, antibody-based imaging, and so on (Uhlén et al., 2015). The default parameters for all these servers were used and the p-value less than 0.05 was considered significant. This analysis enabled a direct comparison of staining intensities between cancerous and non-cancerous tissues.

### 2.3 Correlation of *CKS1B* overexpression with clinical features and promoter methylation

The UALCAN server was used to determine the correlation of *CKS1B* overexpression with clinical features (Chandrashekar et al., 2017). Individual cancer stages, patient’s race, patient’s gender, patient’s age, patient’s smoking habit, tumor histology, nodal metastasis status, and *TP53* mutation status are the clinical features that were analyzed in the server keeping all the parameters default. After that, the UCSC Xena Functional Genomic Explorer (https://xenabrowser.net/) was used to find out the DNA methylation pattern of *CKS1B* gene promoter associated with LUAD and LUSC (Goldman et al., 2020). The TCGA server analyzed experimental data of 706 LUAD and 626 LUSC samples, and the DNA Methylation of 27K and 450K patterns of *CKS1B* for LUAD and LUSC respectively. Statistical significance (p-value <0.05) was determined for each correlation.

### 2.4 Analyses for mutations and copy number alterations

The pattern of mutations and copy number alterations of the *CKS1B* gene in association with LUAD and LUSC was determined using the cBioPortal server (https://www.cbioportal.org/; designed to explore, visualize, and interpret multidimensional cancer genomics data) (Cerami et al., 2012). This platform was utilized to investigate DNA methylation levels in the promoter region of CKS1B using data from Illumina Human Methylation 450K and 27K arrays. Differential methylation patterns between LUAD and LUSC were assessed to identify potential epigenetic mechanisms influencing gene expression. In this study, this platform provided an overview of mutations and copy number variations (CNVs) in *CKS1B* across 2,983 LUAD and 1,176 LUSC samples. We categorized these alterations by frequency and type, including missense mutations, amplifications, and deep deletions, to assess their potential contributions to lung cancer pathogenesis. The mutation spectrum of the *CKS1B* gene responsible for cancer development was determined by the cBioPortal server, keeping all the parameters as default.

### 2.5 Analysis of correlation between *CKS1B* expression and the survival of LC patients

PrognoScan server (http://dna00.bio.kyutech.ac.jp/PrognoScan/) was exploited to determine the relationship of the survival of patients with LUAD and LUSC and *CKS1B* gene expression. PrognoScan is a database for meta-analysis of genes that scans the freely available microarray datasets to predict the interaction between the expression of a query gene and the prognosis of cancer (Mizuno et al., 2009). The server uses the Kaplan-Meier statistical method to plot the gene expression in the X-axis and the possibility of patient survival in the Y-axis. During the analysis, default parameters were used and any Cox p-value less than 0.05 was considered to be statistically significant. Datasets with significant associations (Cox p-value <0.05) were included to determine the relationship between *CKS1B* expression and overall survival (OS) and relapse-free survival (RFS).

### 2.6 Analysis of the genes co-expressed with *CKS1B* in LC tissues

Three different servers i.e., Oncomine (Rhodes et al., 2004), GEPIA2 (Tang et al., 2019), and UCSC Xena web browser (Goldman et al., 2020) were used to determine the genes which tend to co-express with *CKS1B*. Firstly, the Oncomine server was used to search the list of co-expressed genes. The server ranks the co-expressed genes based on correlation scores. Thereafter, the analysis of the relationship between the *CKS1B* gene and the gene that generated the highest correlation score in the Oncomine server was carried out using the GEPIA2 server. Finally, the UCSC Xena web browser was utilized to design the gene expression pattern of the selected genes in LUAD and LUSC patients.

### 2.7 Network and pathway analysis in LUAD and LUSC for the *CKS1B* gene

GeneMANIA (https://genemania.org) is a web-based platform that uses a large database of functional interaction data to assess the relationship between a gene of interest and other genes. Centered on protein and genetic interactions, pathways, co-expression, co-localization, and protein domain similarity, this platform was used to construct an interaction network of *CKS1B* and related gene. In addition, using the International Molecular Exchange Consortium (IMEx) protein interactions database, the previously reported 20 associated genes and *CKS1B* was used in NetworkAnalyst (https://www.networkanalyst.ca/) to establish the protein-protein interaction at a generic level. The RTK/Ras pathway, P13K/AKT pathway, MAPK, and Rap1 signaling pathway were found to involve *CKS1B* in the KEGG 2019 database. Following that, PathwayMapper in the cBioPortal server was used to analyze these pathways in detail as well as to estimate the frequency of *CKS1B* alterations.

### 2.8 Determination of *CKS1B* ontology and signaling pathways of cancer-associated genes

The Enrichr server (http://amp.pharm.mssm.edu/Enrichr/) was used for gene ontology (GO) and cell signaling pathways of *CKS1B*. The server generates the annotation enrichment results of a target gene set by comparing multiple genomics datasets containing previous biological knowledge (Chen et al., 2013; Kuleshov et al., 2016). To do the analysis, the *CKS1B* along with the other co-expressed genes found in the Oncomine server was used. The GO terms (i.e., GO biological process, GO molecular function, and GO cellular component) and the signaling pathways (from BioPlanet 2019; KEGG 2019 Human, and Reactome 2016 databases) were analyzed using the Enrichr web server. Validation was performed across multiple databases to enhance reproducibility and reliability.

## 3 Results

### 3.1 Expression of *CKS1B* mRNA in cancerous and non-cancerous tissues

**FIGURE 1:**
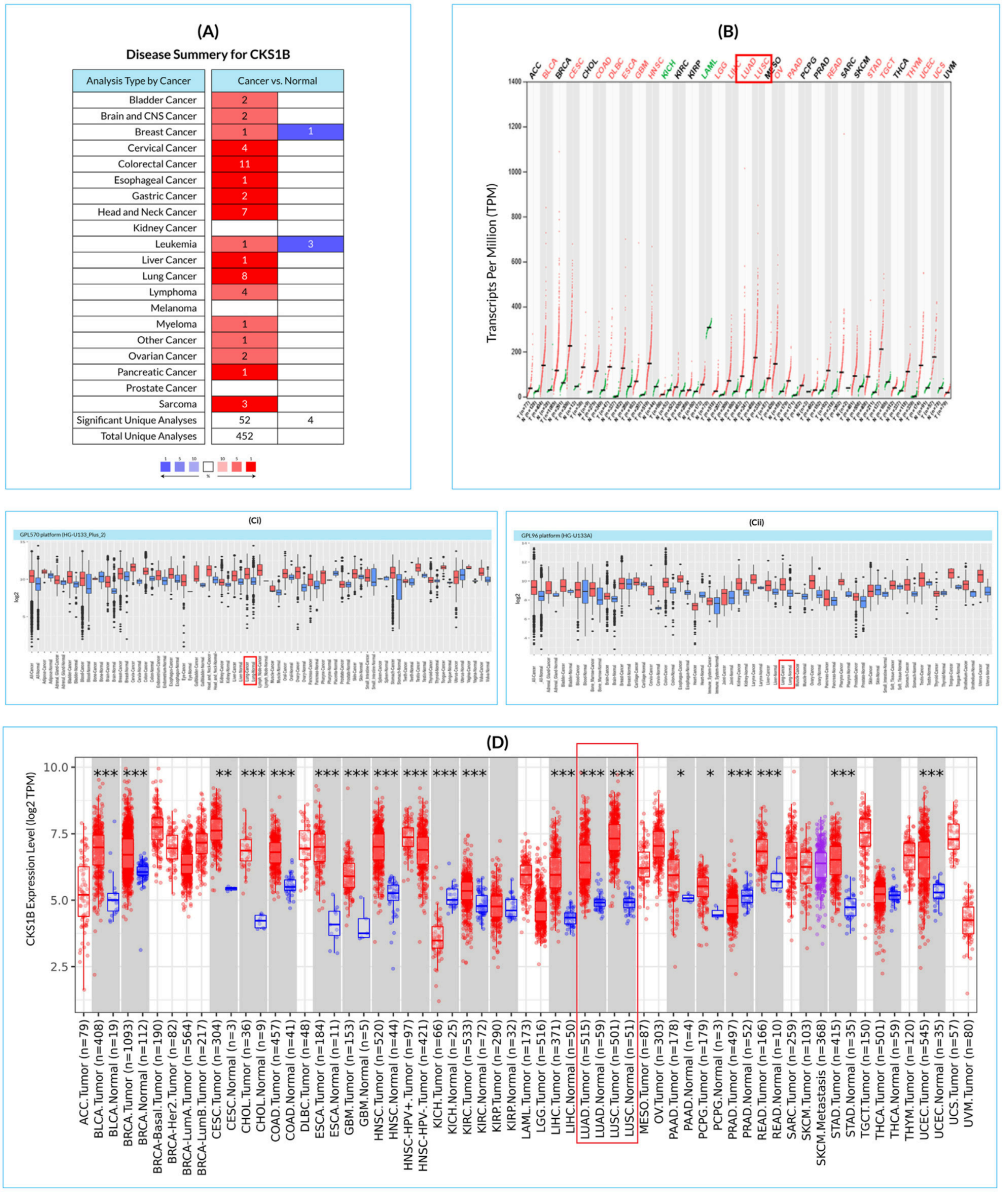
*CKS1B* tissue-wide expression profile across multiple cancer types in humans. **(A)** Disease summary heatmap displaying differential expression of *CKS1B* across various cancers. Red denotes higher expression in cancer tissues compared to normal tissues, while blue indicates lower expression. **(B)** Dot plot illustrating the gene expression profile of *CKS1B* in 33 human cancers, including both tumor and healthy tissue samples. **(C)** Box plots representing *CKS1B* mRNA expression in cancerous and corresponding healthy tissues, using the Affymetrix HG-U133Plus 2 **(Ci)** and HG-U133A **(Cii)** platforms from the GENT2 database. Boxes show the median, dots represent outliers, and red boxes indicate cancerous tissues, while blue boxes represent healthy tissues. **(D)** Differential expression of *CKS1B* mRNA in LUAD and LUSC tissues relative to normal tissues (*P < 0.05; **P < 0.01; ***P < 0.001, based on differential analysis).

*CKS1B* is substantially upregulated in various cancer cells, including LC, according to 56 studies out of 452 retrieved datasets (Figure 1A). GEPIA2 results also indicate a similar pattern of *CKS1B* upregulation in 33 different types of cancer cells including LUAD and LUSC (Figure 1B). *CKS1B* is overexpressed in various cancers, including LUAD and LUSC, according to a review of GENT2 databases using HG-U133pLUS 2 and HG-U133A platforms (Figure 1Ci, Cii). In addition, the TIMER analysis revealed that *CKS1B* was overexpressed in both LUAD and LUSC as compared to other cancer forms (Figure 1D). Overall, the findings showed that the *CKS1B* gene is significantly upregulated in LC tissues as compared to normal tissues and other cancer cells (Figure 1).

### 3.2 Expression of *CKS1B* transcript in human LC tissues

**FIGURE 2:**
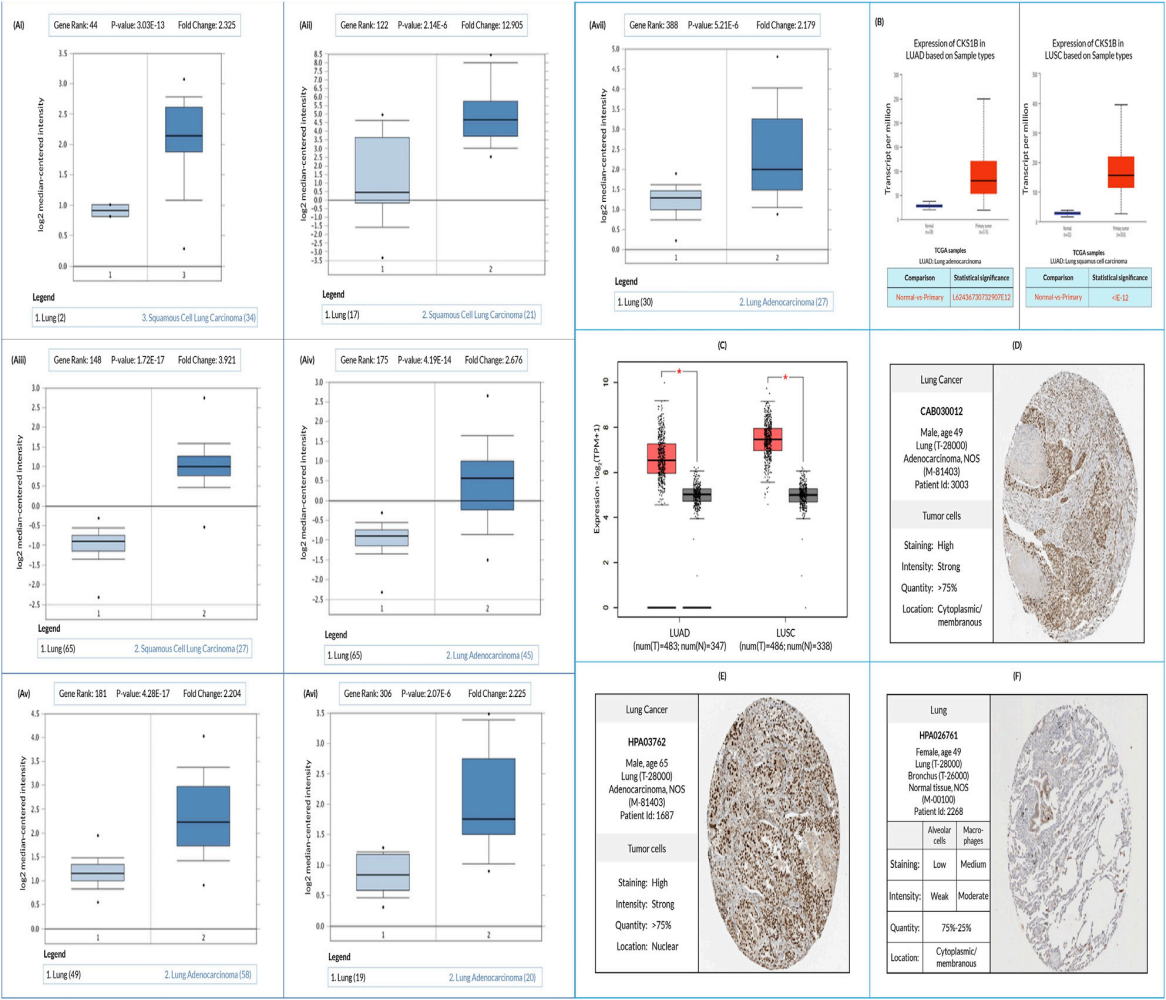
Evaluation of *CKS1B* expression in LUAD and LUSC. **(A)** Box plots comparing *CKS1B* expression between normal (left) and cancerous (right) tissues in LUAD and LUSC **(Ai–Avii)**. **(B)** Box plots showing *CKS1B* mRNA expression in tumor and normal tissues for LUAD and LUSC using the UALCAN platform. **(C)** *CKS1B* mRNA expression in LUAD and LUSC analyzed using GEPIA2. **(D–F)** Immunohistochemistry images showing *CKS1B* expression in LUAD **(D)**, LUSC **(E)**, and normal tissues **(F)** from the HPA database.

Comparative analysis using Oncomine server between LC (LUAD and LUSC) and normal tissue revealed an increased expression of the *CKS1B* gene in both LC subtypes (Figure 2Ai-vii; Supplementary Table S1). Additional evaluation of TCGA datasets using the UALCAN and GEPIA2 server also represented a significant overexpression of *CKS1B* in the LC subtypes (Figures 2B, C). Moreover, relative immunohistochemistry analysis of healthy and cancerous tissues using the HPA database showed moderate to weak staining signals in normal alveolar cells (Figure 2F), and strong signals in both LUAD (Figure 2D) and LUSC (Figure 2E) tissue samples (Figure 2; Supplementary Table S1).

### 3.3 Correlation between *CKS1B* expression and clinical features of LC patients

**FIGURE 3:**
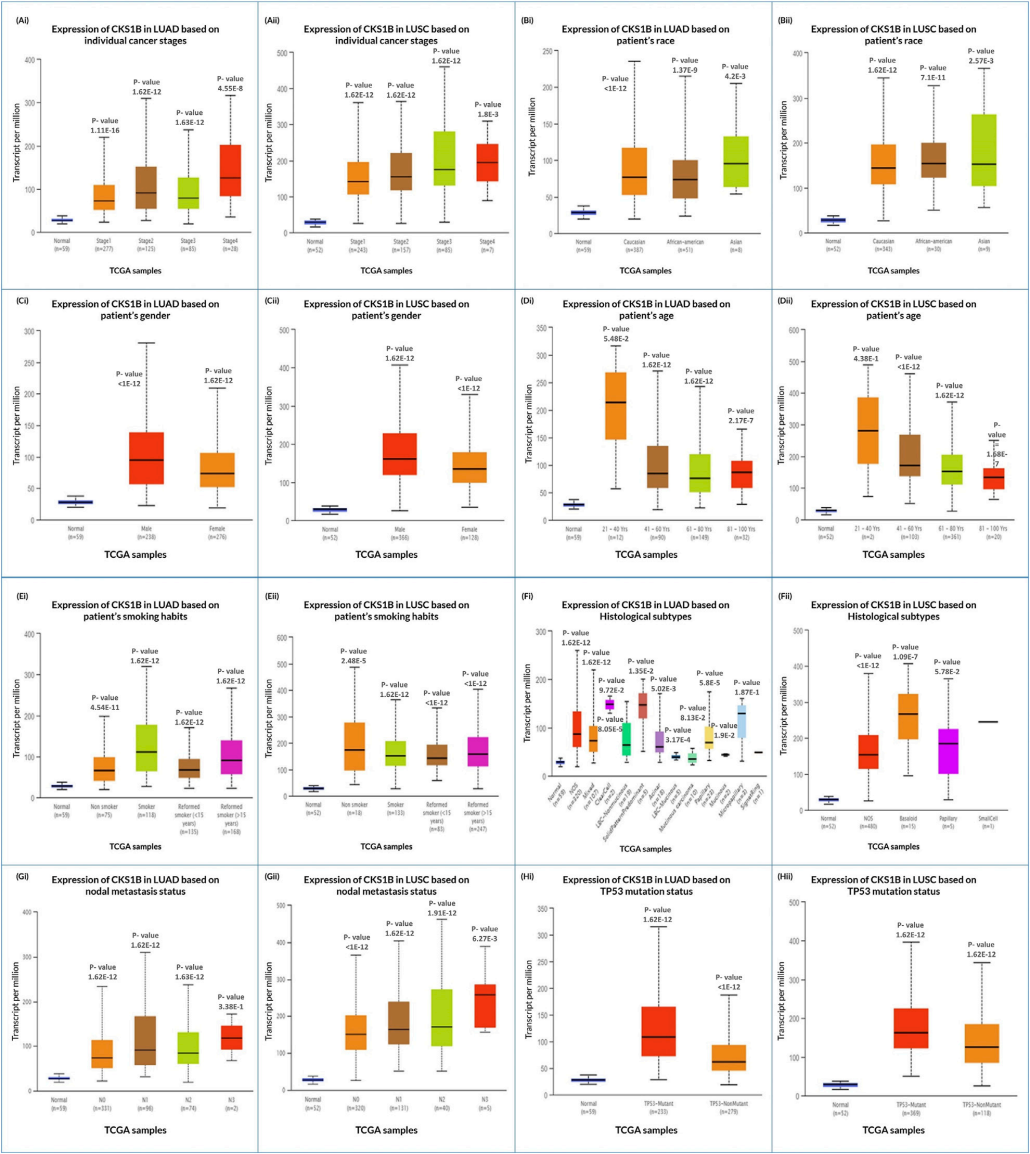
Correlation of *CKS1B* expression with diagnostic criteria in LUAD and LUSC patients. *CKS1B* mRNA expression is analyzed across individual cancer stages, race, gender, age group, smoking habits, histological subtypes, nodal metastasis, and TP53 status for **(Ai–Hi)** LUAD and **(Aii–Hii)** LUSC.

Analysis of the UALCAN database revealed that *CKS1B* upregulation for LUAD and LUSC varied in individual cancer stages (Figures 3Ai, ii) due to patient ethnicity, sex, age, smoke addiction, tumor histology, nodal metastasis, and TP53 mutation status (Figure 3; Supplementary Tables S2, S3). Correlated overexpression of *CKS1B* was also found in the patient of Asian (Figures 3Bi, ii), male (Figures 3Ci, ii), age group range of 21–40 years old (Figure 3Di, ii), smoking habit (Figures 3Ei, ii), tumor histology (Figures 3Fi, ii), nodal metastasis (Figures 3Gi, ii), and TP53 mutation status (Figures 3Hi, ii), for both LUAD and LUSC (Supplementary Tables S2, S3). These findings suggest a potential correlation of *CKS1B* upregulation and clinical characteristics with LC than normal patients.

### 3.4 TCGA dataset utilization for analysis of LC promoter methylation

**FIGURE 4:**
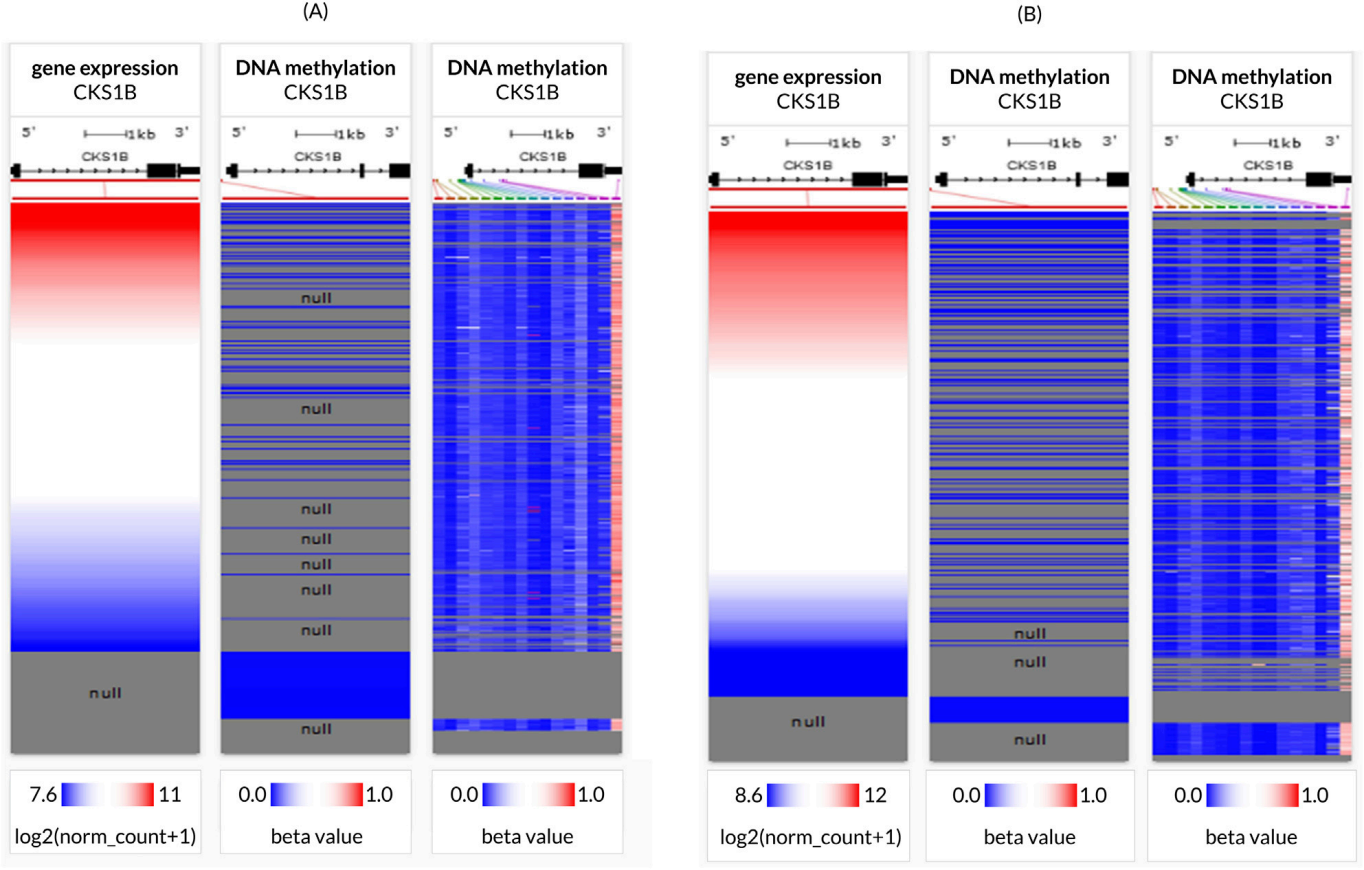
*CKS1B* gene promoter methylation in lung cancer tissues. Heatmaps illustrate the *CKS1B* expression and DNA methylation status in **(A)** LUAD and **(B)** LUSC tissues.

Association between *CKS1B* expression and DNA methylation was evaluated using two separate methylation patterns present in the server, namely, Human Methylation 27K and Human Methylation 450K for both the LC subtypes (Figures 4A, B). Retrieved heat maps showed a close negative correlation between *CKS1B* expression and some CpG islands in LUAD (Figure 4A), whereas LUSC (Figure 4B) suggested that the overexpression of *CKS1B* associated with decreased DNA methylation in LUAD and LUSC, and *vice versa* (Figure 4).

### 3.5 Analyzing mutations, copy number alterations, and expression of mutant *CKS1B* transcripts

**FIGURE 5:**
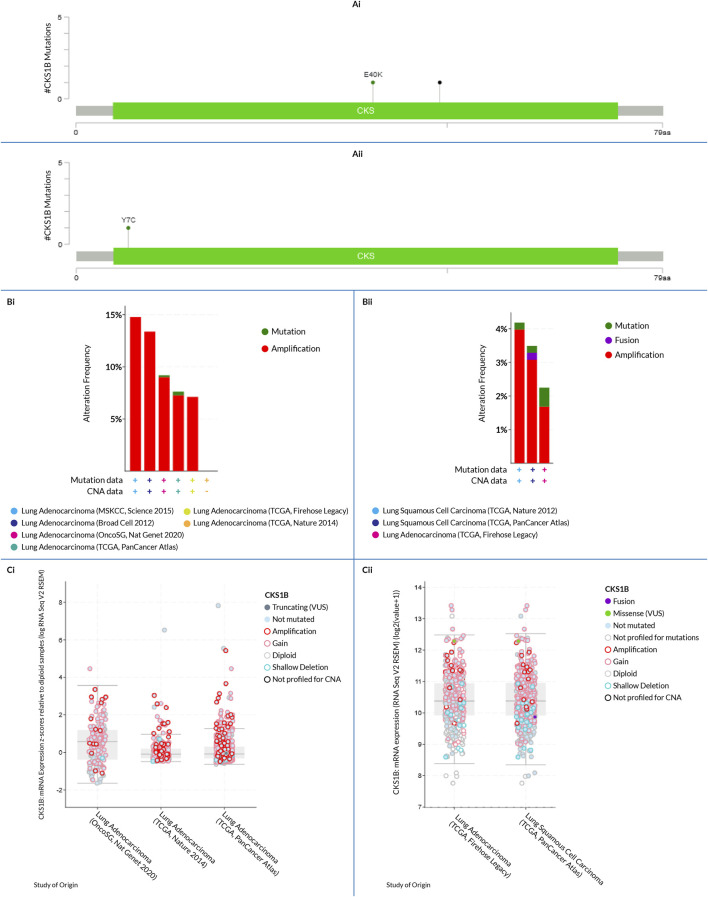
*CKS1B* gene alterations and mutations in LUAD and LUSC tissues. **(Ai)** Lollipop plot showing mutation spots in the CKS domain of *CKS1B* in LUAD tissues (residues 40–50). **(Aii)** Lollipop plot showing mutation spots in *CKS1B* in LUSC tissues. **(Bi, Bii)** Bar diagrams depicting mutation frequencies and genome alterations in *CKS1B* for LUAD and LUSC, respectively. **(Ci, Cii)** Correlation between *CKS1B* expression and copy number alterations for LUAD and LUSC in the TCGA dataset.

An analysis of the *CKS1B* gene alterations in both the subtypes of LC was done using the cBioPortal database; totaling 2,983 samples from 8 LUAD studies and 1,176 samples from 3 LUSC studies (Table 1). Studies revealed 191 (6%) alteration in the *CKS1B* gene in LUAD samples (Figure 5Ai), with a somatic mutation frequency of 0.1 percent and 42 (4%) altered *CKS1B* gene in LUSC samples with a somatic mutation frequency of 0.3 percent (Figure 5Aii). *CKS1B* is found between 154,974,681 and 154,979,251 bp on chromosome 1q21.3. (http://atlasgeneticsoncology.org/Genes/CKS1BID40092ch1q21.html). Two mutations including one missense and one nonsense were found between 40–50 residues of the CKS domain of the 79 AA long *CKS1B* protein in LUAD samples (Figure 5Ai) whereas three missense mutations in the same mutation site (7th residue), as well as one fusion, were found in LUSC samples (Figure 5Aii). Furthermore, as seen in Figure 5Bi, amplification was found to be the most prevalent alteration type in both LC subtypes, with the highest frequency of 14.78% of 230 cases in the LUAD TCGA, Nature 2014 dataset. The LUSC TCGA, Firehose Legacy dataset had the highest alteration frequency of the three, at 4.18% of 502 cases. In the alteration study of LUAD TCGA, Nature 2014 dataset, both mutation and amplification of *CKS1B* have been identified with a frequency of 0.2% (1 case) and 3.98% (20 cases), respectively. In the LUSC TCGA, PanCancer Atlas, a fusion was also observed with a frequency of 0.21% (Figure 5Bii). Additionally, the *CKS1B* mRNA expression profile exhibited higher amplification, fewer gains, and twelve cases of shallow deletions in LUAD samples (Figure 5Ci). In LUSC samples, two missense mutations and one fusion along with some gains, diploids, and shallow deletions were found, as appeared in Figure 5Cii.

**TABLE 1:** Selected *CKS1B* mutational positions and types in LUAD and LUSC from the TCGA dataset.

Sample ID	Cancer type	Protein change	Mutation type	Chromosome No	Start position	End position	Number of samples
TCGA-55-8614-01	Lung Adenocarcinoma	Q49*	Nonsense	1	154,950,548	154,950,548	203
BGI-RS55	Lung Adenocarcinoma	E40K	Missense	1	154,950,521	154,950,521	107
TCGA-66-2758-01	Lung Squamous Cell Carcinoma	Y7C	Missense	1	154,947,241	154,947,241	278
TCGA-66-2758-01	Lung Squamous Cell Carcinoma	Y7C	Missense	1	154,947,241	154,947,241	289
TCGA-66-2758-01	Lung Squamous Cell Carcinoma	Y7C	Missense	1	154,947,241	154,947,241	267
TCGA-85-A50Z-01	Lung Squamous Cell Carcinoma	EFNA3-*CKS1B*	Fusion	1	−1	−1	397

Consequently, the observations suggest that *CKS1B* overexpression in LUSC and LUAD may not have an established correlation with mutations or copy number alterations in the *CKS1B* gene (Figure 5). Nevertheless, these genetic modifications can serve as prognostic measures for LUAD and LUSC, using *CKS1B* as a biomarker. Also, the correlation of *CKS1B* to other functional proteins which are important for cell cycle, proliferation, and DNA repair, makes it a key component for increasing human susceptibility to diseases or detrimental effects, in case of mutations or alterations. This raises the importance of thorough future evaluations in regards to mutations and copy number alterations.

### 3.6 Correlation between *CKS1B* expression and clinical prognosis of LC patients

**FIGURE 6:**
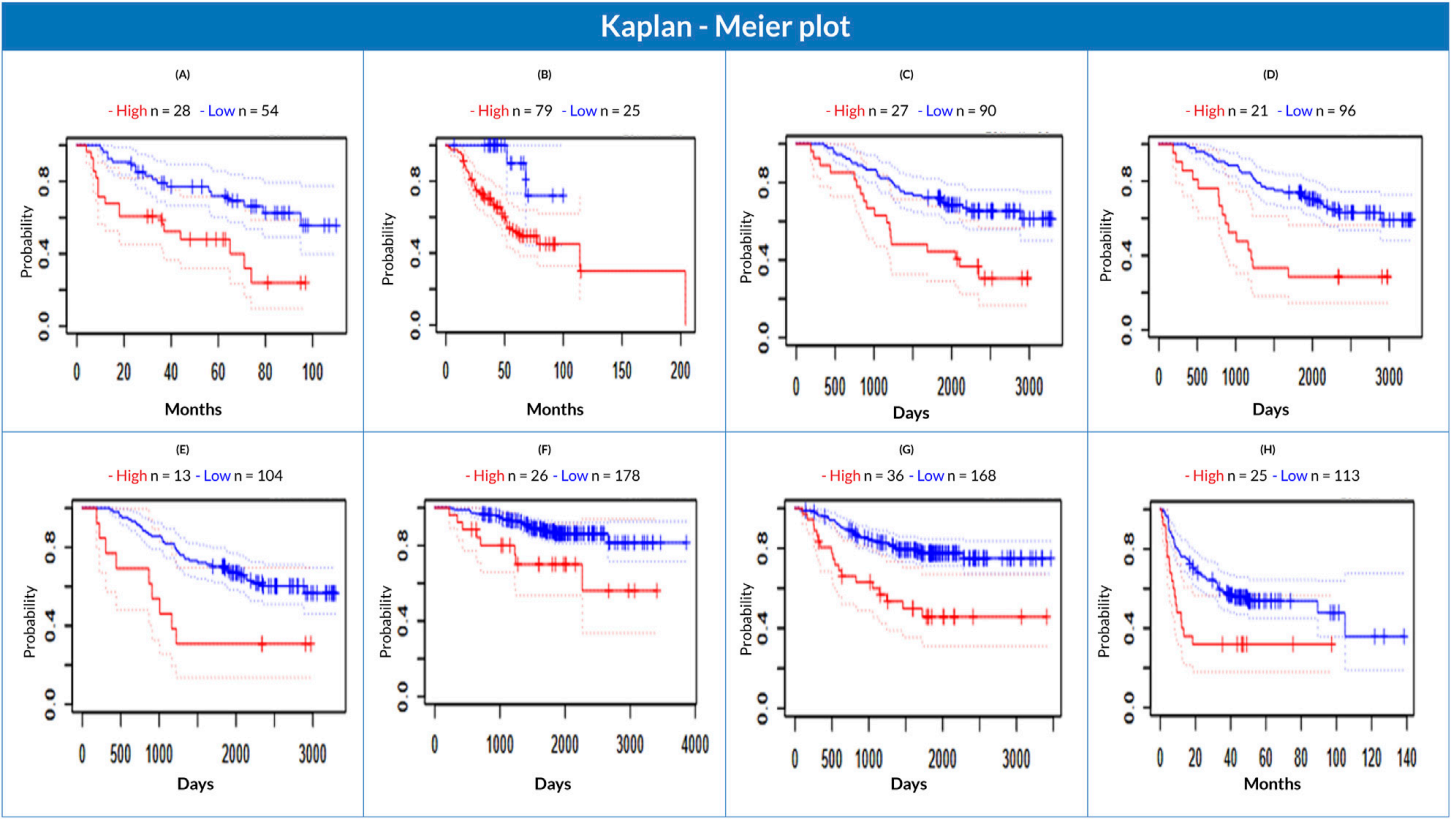
Correlation of *CKS1B* expression with lung cancer patient survival. Kaplan-Meier survival plots comparing high (red) and low (blue) *CKS1B* expression for overall survival **(A–F)** and relapse-free survival **(G, H)**.

A negative correlation between *CKS1B* expression and LC patient survival (significant level was held at Cox p-value 0.05 and HR > 1) was found using the PrognoScan database. *CKS1B* expression was observed to be negatively associated with patient survival. This finding indicated that patients with elevated *CKS1B* expression might have a lower survival risk, whereas those with poor or average *CKS1B* expression may have a higher survival (overall or relapse-free) rate (Figure 6; Supplementary Table S4). The datasets showed an increased overall survival risk in patients with low *CKS1B* expression, while those with higher *CKS1B* expression had a considerably low overall survival rate (Figure 6). The investigation suggests a correlation between increased *CKS1B* expression and a poor prognosis in LC patients.

### 3.7 Determination of *CKS1B* and human LC associated gene signatures

**FIGURE 7:**
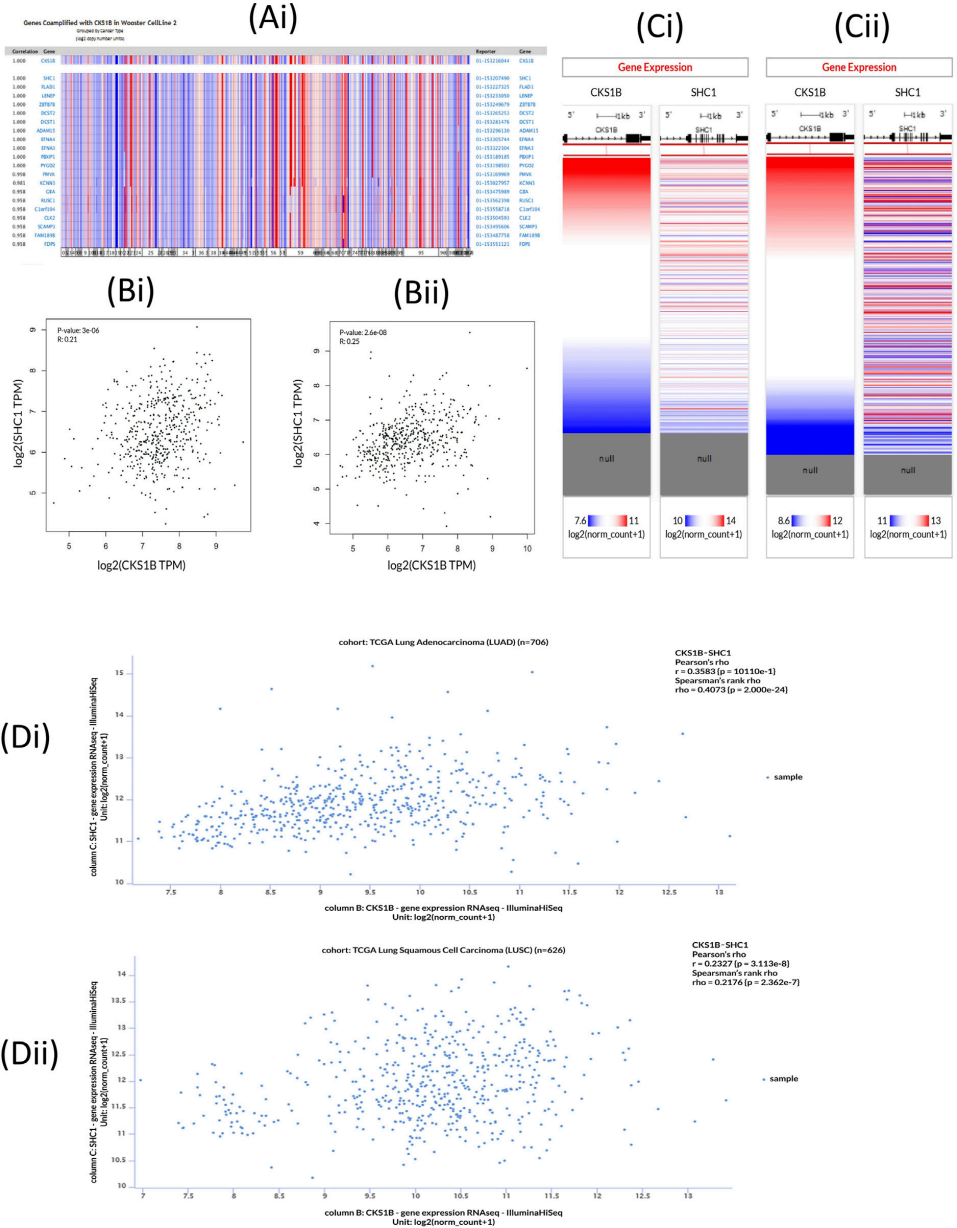
*CKS1B* and co-expressed gene profiles in lung cancer. **(Ai)** Co-expression profile of *CKS1B* retrieved from the Oncomine database. **(Bi-ii)** Correlation analysis between *CKS1B* and *SHC1* from the GEPIA2 server. **(Ci-ii)** Heatmap showing mRNA expression of *CKS1B* and *SHC1* from the TCGA database. **(Di-ii)** Co-expression analysis of *CKS1B* and *SHC1* in lung cancer using the UCSC Xena server.

The Oncomine database was utilized to investigate the co-expression profile of *CKS1B* with 20 other genes, using 34 samples from LUAD and LUSC patients (Figure 7A). Among the 20 genes analyzed, the most co-expressed (R = 1.00) was the Src homology 2 domain-containing transforming protein C1 *(SHC1)*. Following this, the GEPIA2 server also indicated a positive correlation with a Spearman coefficient in both LUAD (R = 0.25) and LUSC (R = 0.21) between *CKS1B* and *SHC1* (Figure 7Bii). This was further supported by Pearson and Spearman correlation analyses results obtained from the UCSC Xena server, using the TCGA database (Figures 7C, D). The Pearson correlation values for LUAD (Figures 7Ci–Di) and LUSC (Figures 7Cii–Dii) patients were 0.3563 and 0.2327, and the Spearman correlation values for LUAD and LUSC were 0.4073 and 0.2176, respectively (Figures 7C, D). These values suggest that *CKS1B* and *SHC1* can share a common biosynthetic pathway, or combine to form a large protein complex with LC progression.

### 3.8 Analysis of *CKS1B* gene network in LUAD and LUSC

**FIGURE 8:**
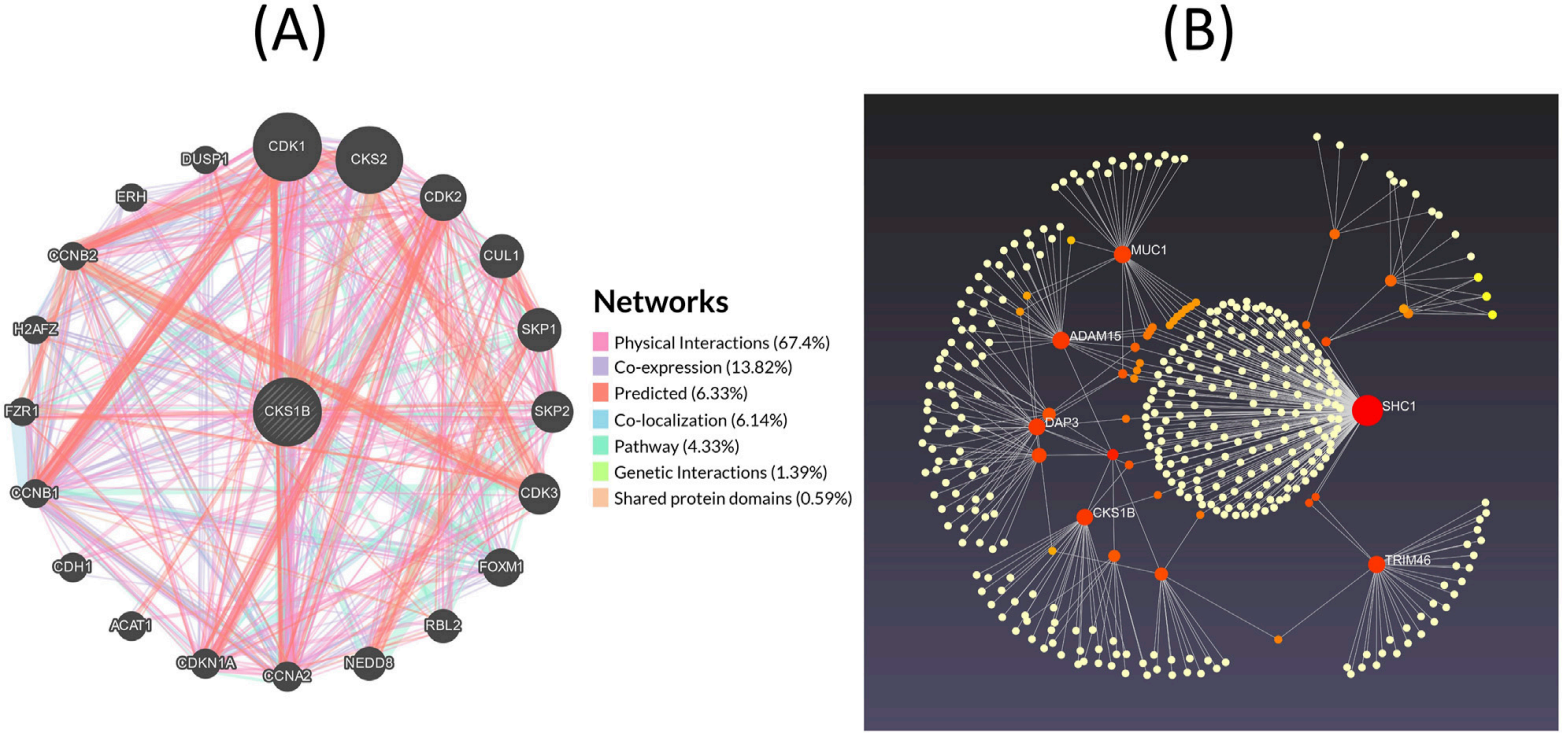
Gene interaction networks for *CKS1B*. **(A)** Interaction network of *CKS1B* with neighboring genes, categorized into physical interactions (67.40%), co-expression (13.82%), predicted (6.33%), co-localization (6.14%), pathways (4.33%), genetic interactions (1.39%), and shared protein domains (0.59%). **(B)** Protein-protein interaction network of *CKS1B* from the IMEx database.

The GeneMANIA server retrieves a complete network of *CKS1B* gene and its crosstalk genes of interaction in LC, displaying the physical interactions (67.40%), co-expression (13.82%), predicted (6.33%), co-localization (6.14%), pathways (4.33%), genetic interactions (1.39%) and shared protein domains (0.59%) (Figure 8A). Moreover, based on the International Molecular Exchange Consortium (IMEx) protein interactions database, protein-protein interaction (PPI) network was constructed for the co-expressed genes, and using NetworkAnalyst, these interactions were further analyzed for LUAD and LUSC progressions. Figure 8B shows the PPI network in which, the degree of a node is the number of connections among the node, and betweenness is the smallest path amongst nodes showing *SHC1* (Degree: 207, Betweenness: 65,895.06), MUC1 (Degree: 31, Betweenness: 7,217.707), ADAM15 (Degree: 31, Betweenness: 9,000.452), TRIM46 (Degree: 30, Betweenness: 10,467.46), DAP3 (Degree: 28, Betweenness: 8,994.579), *CKS1B* (Degree: 27, Betweenness: 9,277.062), and *ZBTB7B* (Degree: 19, Betweenness: 6,702.692) as the major proteins of the network.

### 3.9 Evaluation of *CKS1B* gene associated pathways in LUAD and LUSC

**FIGURE 9:**
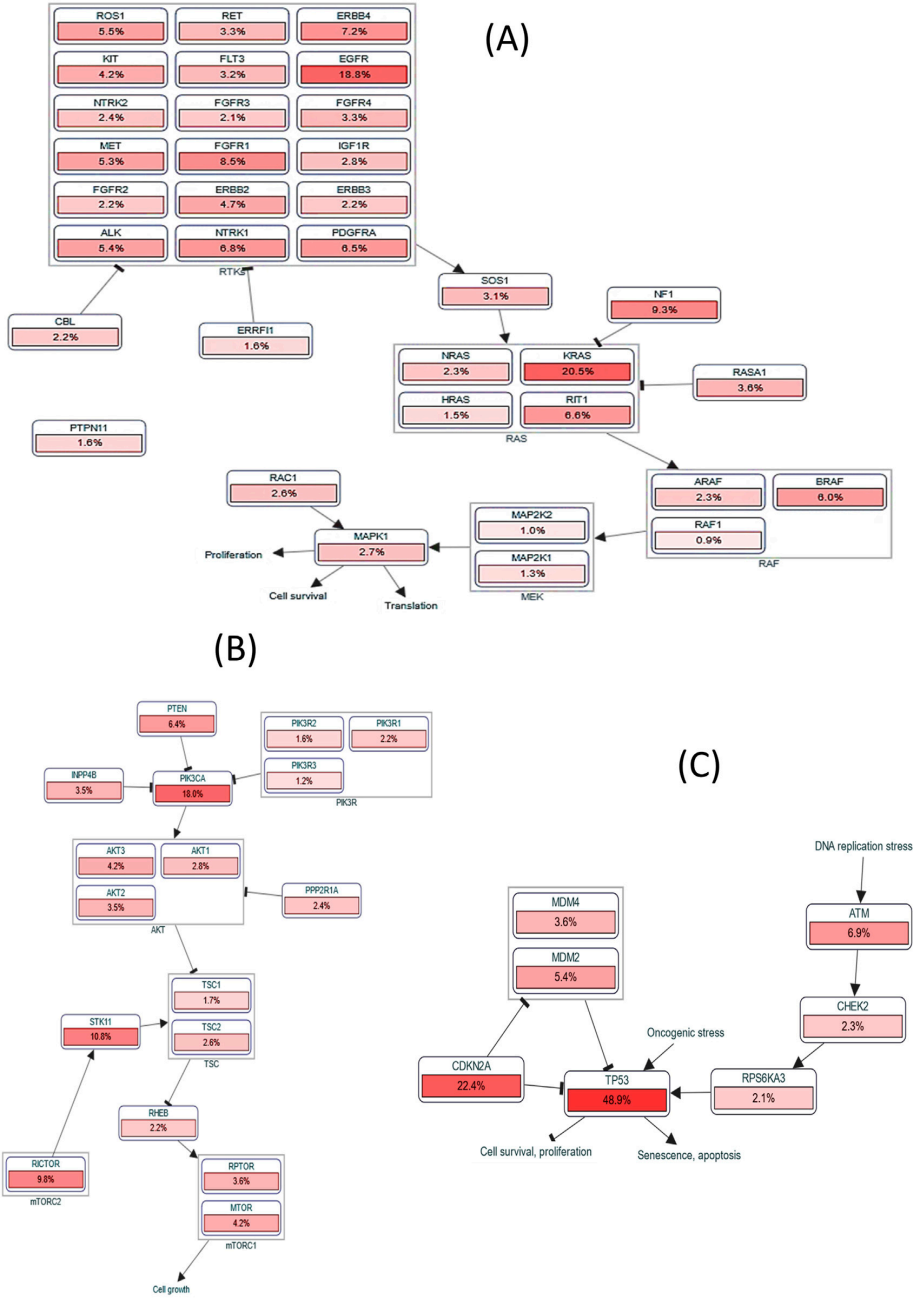
Pathway analysis for *CKS1B* and related genes. Alteration frequency in **(A)** RTK-Ras-RAF signaling pathway, **(B)** P13K/AKT signaling pathway, and **(C)** TP53 pathway in relation to *CKS1B* expression.

The PathwayMapper tab in the cBioPortal server exhibits the *CKS1B* alteration frequency (in percentage) over multiple pathways on the LUAD and LUSC datasets (Figures 9A, B). RTK-Ras-RAF, PI3K/AKT, and TP3 pathways are mainly altered due to *CKS1B* gene products. These alterations coordinate the progression of LUAD and LUSC. Alterations related to *CKS1B* mainly induce the changes of EGFR (18.8%), KRAS (20.5%), FGFR1 (8.5%), and BRAF (6.0%) genes for regulation of RTK-Ras signaling pathway (Figure 9A) as well as PTEN (6.4%), PIK3CA (18.0%), STK11 (10.8%), and RICTOR (9.8%) in the regulation of P13K/AKT signaling pathway (Figure 9B); TP53 (48.9%), and CDKN2A (22.4%) in TP53 signaling pathway (Figure 9C) for cancer proliferation and development.

### 3.10 Identification of *CKS1B* ontologies and related signaling pathways involved in LC

**FIGURE 10:**
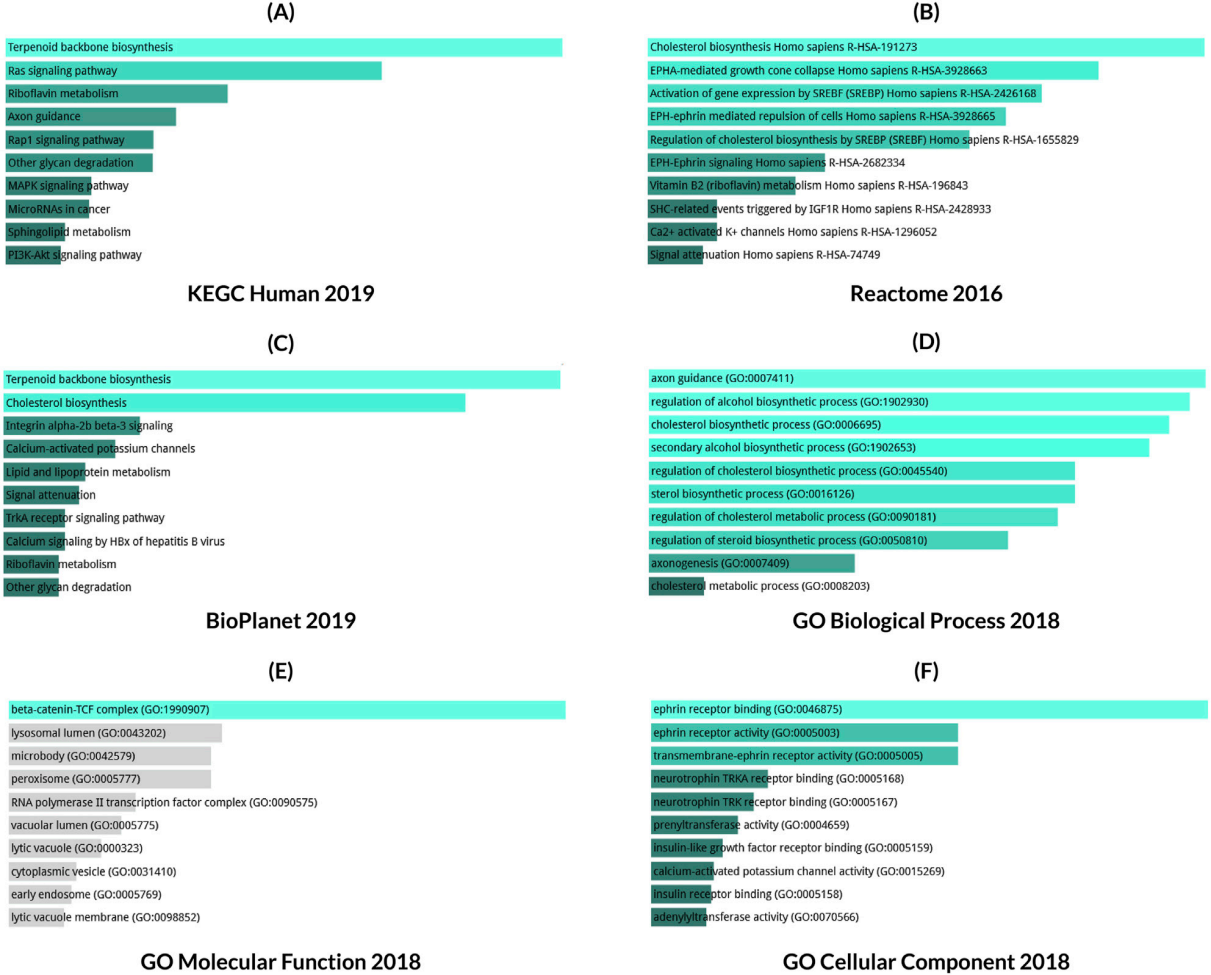
Gene ontology and pathway enrichment analysis for *CKS1B* in lung cancer. Ontologies and pathways retrieved from **(A)** KEGG human 2019, **(B)** Reactome 2016, **(C)** BioPlanet 2019, **(D)** GO Biological Process 2018, **(E)** GO Molecular Function 2018, and **(F)** GO Cellular Component 2018. Bar length represents significance level, with brighter colors indicating higher significance.

Results from three databases shown in Figures 10A–C were used for pathway determination. The KEGG human 2019 database analysis revealed different significant associations of different pathways in LC progression including RTK/Ras pathway, P13K/AKT pathway, MAPK, and Rap1 signaling pathway (Figure 10A). Additionally, the Reactome 2016 database showed LC progression depends on the pathways related to EPHA or EPH-Ephrin mediated signaling and repulsion of the cells, etc. (Figure 10B). The analysis of the Bioplanet 2019 database also revealed the Ephrin receptor A forward pathway, and p27 phosphorylation regulation during cell cycle progression alongside other important pathways associated with LC (Figure 10C). Following this, GO terms were also checked for the corresponding genes which mainly include, positive regulation of aspartic-type peptidase activity, axon guidance and axogenesis (Figure 10D), ephrin receptor binding and transmembrane-ephrine receptor activity (Figure 10F), and possible interactions with beta-catenin-TCF complex, mitochondrial small ribosomal subunit, and an anchored component of the plasma membrane (Figure 10E).

## 4 Discussion

Lung cancer (LC) remains the leading cause of cancer-related mortality worldwide (Cancer Genome Atlas Research Network, 2014). NSCLC consisting of both LUAD and LUSC solely constitutes approximately 85% of all LC cases. LC is notoriously difficult to diagnose at an early stage, with most patients (about 75%) being diagnosed in advanced stages (stage III/IV), highlighting the need for early detection (Walters et al., 2013). Early diagnosis is crucial for better prognosis and survival rates, as shown by the significantly higher 5-year survival rates (70%–90%) in patients with stage I NSCLC following surgical resection (Shah et al., 1996; Nesbitt et al., 1995; Goldstraw et al., 2016). Despite advances in treatment, early detection remains the key to improving survival outcomes (Bradley et al., 2019).

In this study, we evaluated the role of *CKS1B* as a potential prognostic biomarker for the early diagnosis of LUAD and LUSC using bioinformatics approaches. Our analysis revealed a positive correlation between *CKS1B* expression levels and LC progression. High *CKS1B* expression was also negatively correlated with overall and relapse-free survival in LUAD and LUSC, as evidenced by an overall hazard ratio (HR) greater than 1. This suggests that elevated *CKS1B* expression is associated with poor survival outcomes, corroborating previous studies linking high *CKS1B* levels to adverse prognosis in LC (Shi et al., 2020b).

Further analysis revealed that *CKS1B* expression patterns in LUAD and LUSC were significantly associated with key clinical features such as tumor histology, patient demographics (e.g., race, gender, age, smoking status), and nodal metastasis (Figure 3). This highlights the importance of comprehensive studies that consider these variables, as *CKS1B* overexpression may serve as a marker of cancer progression (Figure 6). The immunohistochemical analysis, enhanced by computer-aided systems and digital imaging, revealed robust nuclear immunoreactivity for *CKS1B* in LUAD and LUSC tissues, with significantly more intense staining observed in cancer cells compared to normal alveolar cells (Figure 2). This suggests elevated *CKS1B* expression in lung cancer cells. Such findings indicate that *CKS1B* plays a pivotal role in the pathogenesis of NSCLC, reflecting the complex histopathological processes driven by somatically acquired genetic, epigenetic, transcriptomic, and proteomic alterations responsible for cancer progression (Laurinaviciene et al., 2012; Laurinavicius et al., 2016; Yu and Tian, 2020b).

On a molecular level, somatic genetic alterations, such as copy number changes and mutations, are known to drive cancer progression. Our analysis using the cBioPortal webserver indicated that the *CKS1B* gene was altered in 6% of LUAD samples and 4% of LUSC samples, with missense mutations being the most common genetic change (Figures 5A–C). This suggests that genetic alterations in *CKS1B* may contribute to LC pathogenesis, although their low frequency highlights the need for further investigation.

Interestingly, we observed a negative correlation between *CKS1B* expression and DNA methylation of CpG islands in gene promoters, a finding consistent with previous studies linking promoter methylation to transcriptional silencing in various cancer types, including LC (Slotky et al., 2005). Additionally, co-expression and correlation analysis identified 20 genes positively correlated with *CKS1B*, with *SHC1* emerging as a key player in NSCLC progression through the Ras/ERK and PI3K/AKT signaling pathways (Figure 7) (Liu et al., 2019). This supports the idea that *CKS1B* and its co-expressed genes may interact in a network that influences tumor cell behavior and malignancy.

Moreover, we explored the functional network of *CKS1B* and its neighboring genes in lung cancer, highlighting significant interactions that include co-localization, pathways, and genetic interactions (Figure 8). *CKS1B* interacts with cyclin-dependent kinases (CDKs) and S-phase kinase-associated proteins (SKPs), suggesting its critical role in regulating the cell cycle. *CDK1,* in particular, is essential for G2/M and G1/S transitions and is often overexpressed in malignancies, which correlates with poor patient prognosis (Santamaria et al., 2007; Malumbres and Barbacid, 2009; Enserink and Kolodner, 2010; Ravindran Menon et al., 2018; Li et al., 2020). Furthermore, *CKS1B* promotes the degradation of the cell cycle inhibitor p27Kip1 via its interaction with *SKP2,* leading to increased cell proliferation (You et al., 2015). This dysregulation of *CKS1B* can impact a broader network of co-expressed genes, which are regulated by shared transcriptional programs, thereby influencing multiple biological pathways (Weirauch, 2011; Melak and Gakkhar, 2015). Additionally, proteins such as *SHC1, MUC1,* and *ADAM15* display high betweenness centrality scores, indicating their importance as mediators in lung cancer progression (Gursoy et al., 2008). These insights underscore the potential of targeting *CKS1B* and its associated pathways for therapeutic strategies in lung cancer.

Our study also highlighted the importance of *CKS1B* in regulating multiple signaling pathways associated with cancer. *CKS1B* overexpression was found to alter the RTK-Ras-RAF and PI3K/AKT/mTOR signaling pathways, both of which are heavily implicated in LUAD and LUSC pathogenesis (Figures 9A, B). Genetic alterations in key components of these pathways, such as *EGFR, BRAF, KRAS, PIK3CA*, and *PTEN*, have been well-documented in NSCLC, and our study suggests that *CKS1B* may play a role in modulating these alterations to drive cancer progression (McCubrey et al., 2007; Desai et al., 2014; Herbst et al., 2018; Campbell et al., 2016; Desai et al., 2016; Beigel et al., 2020; Saini et al., 2016; Bethune et al., 2010; Tan, 2020; Yuan and Cantley, 2008; Scheffler et al., 2015). Additionally, the *TP53* pathway, a critical regulator of tumor suppression, was found to be downregulated in connection with *CKS1B* expression, further emphasizing its role in LC progression (Figure 9C).

Finally, pathway and gene ontology (GO) enrichment analyses reinforced the significance of *CKS1B* and its co-expressed genes in multiple oncogenic processes. Notably, ephrin-mediated signaling, aspartic-type peptidase activity (involving Napsin A), and the TCF/β-catenin complex were implicated in LC development, suggesting that *CKS1B* and its associated genes influence diverse molecular pathways in LC (Pasquale, 2010; Ueno et al., 2008; Gao et al., 2018) (Figure 10).

Overall, our study provides substantial evidence supporting the role of *CKS1B* as a prognostic biomarker for early diagnosis in LUAD and LUSC. Although these findings are promising, further *in vitro* and *in vivo* studies are necessary to validate *CKS1B*’s clinical application in NSCLC diagnosis and treatment.

## 5 Conclusion

LC is the predominant cause of cancer-related deaths worldwide. This study aimed to identify the molecular signatures involved in the development and progression of LUAD and LUSC. Cancer prognostic factors are essential for early diagnosis and effective treatment, reducing the risk of overtreatment for patients. *CKS1B* appears to be involved in both LUAD and LUSC, according to our findings. The study further points to the possibility of using *CKS1B* as a biomarker for early LC detection and medication screening in treatment strategies. Furthermore, since *CKS1B* is involved in several important signaling pathways, inhibiting it may be a new way to prevent or reduce LC. *CKS1B* and its expression in LC progression were also linked to potential signaling mechanisms and gene ontological characteristics, according to the study. Researchers could be able to stop cancer from spreading by working on these pathways. As a result, *CKS1B* has been suggested as a useful biomarker and potential therapeutic target for human LC regulation or prevention.

## Data Availability

The original contributions presented in the study are included in the article/Supplementary Material, further inquiries can be directed to the corresponding author.
