# Supplementary material for: Exploring the oncogenic role and prognostic value of CKS1B in human lung adenocarcinoma and squamous cell carcinoma
Source: Front Genet. 2025 Mar 17;16:1449466. doi: 10.3389/fgene.2025.1449466 (PMC11955630; doi:10.3389/fgene.2025.1449466)
Supplement: Supplementary file 1 [file Table1.docx]

**Supplementary Materials**

**Supplementary Table S1.** Expression of *CKS1B* in LUAD and LUSC retrieved from the Oncomine database.

| **Dataset** | **Parameters** | **Samples** | **P- value** | **Gene Rank** | **Fold Change** | **Figure No.** |
| --- | --- | --- | --- | --- | --- | --- |
| Talbot Lung (n= 93) | Normal | 2 | - | - | - | Figure 2 Ai |
|  | Lung Squamous Cell carcinoma | 34 | 3.03E-13 | 44 | 2.325 |  |
| Bhattacharjee Lung (n= 203) | Normal | 17 | - | - | - | Figure 2 Aii |
|  | Lung Squamous Cell carcinoma | 21 | 2.14E-6 | 122 | 12.905 |  |
| Hou Lung (n= 156) | Normal | 65 | - | - | - | Figure 2 Aiii |
|  | Lung Squamous Cell carcinoma | 27 | 1.72E-17 | 148 | 3.921 |  |
|  | Normal | 65 | - | - | - | Figure 2 Aiv |
|  | Lung Adenocarcinoma | 45 | 4.19E-14 | 175 | 2.676 |  |
| Landi lung  (n= 107) | Normal | 49 | - | - | - | Figure 2 Av |
|  | Lung Adenocarcinoma | 58 | 4.28E-17 | 181 | 2.204 |  |
| Stearman Lung (n= 39) | Normal | 19 | - | - | - | Figure 2 Avi |
|  | Lung Adenocarcinoma | 20 | 2.07E-6 | 306 | 2.225 |  |
| Su Lung  (n= 93) | Normal Lung | 30 | - | - | - | Figure 2 Avii |
|  | Lung Adenocarcinoma | 27 | 5.21E-6 | 388 | 2.179 |  |

**Supplementary Table S2**. Correlation between *CKS1B* and selected clinicopathological features of LUAD.

| ***CKS1B* expression based on** | **Features** | **Expression of mRNA** | **Number of samples** | **Statistical significance (p-value)** |
| --- | --- | --- | --- | --- |
| Individual cancer stages | Normal | Underexpression | 59 | - |
|  | Stage-1 | Overexpression | 277 | 1.11022302462516E-16 |
|  | Stage-2 | Overexpression | 125 | 1.62447832963153E-12 |
|  | Stage-3 | Overexpression | 85 | 1.62503344114384E-12 |
|  | Stage-4 | Overexpression | 28 | 4.54549999862053E-08 |
| Patient’s race | Normal | Underexpression | 59 | - |
|  | Caucasian | Overexpression | 387 | <1E-12 |
|  | African American | Overexpression | 51 | 1.37540001610148E-09 |
|  | Asian | Overexpression | 8 | 4.201200E-03 |
| Patient’s gender | Normal | Underexpression | 59 | - |
|  | Male | Overexpression | 238 | <1E-12 |
|  | Female | Overexpression | 276 | 1.62436730732907E-12 |
| Patient’s age | Normal | Underexpression | 59 | - |
|  | 21-40 Yrs | Overexpression | 12 | 5.488400E-02 |
|  | 41-60 | Overexpression | 90 | 1.62436730732907E-12 |
|  | 61-80 | Overexpression | 149 | 1.62447832963153E-12 |
|  | 81-100 | Overexpression | 32 | 2.16600000046974E-07 |
| Patient’s smoking habit | Normal | Underexpression | 59 | - |
|  | Non smoker | Overexpression | 75 | 4.54499771151973E-11 |
|  | Smoker | Overexpression | 118 | 1.62447832963153E-12 |
|  | Reformed Smoker 1 | Overexpression | 135 | 1.62447832963153E-12 |
|  | Reformed Smoker 2 | Overexpression | 168 | 1.62458935193399E-12 |
| Histological subtypes | Normal | Underexpression | 59 | - |
|  | NOS | Overexpression | 820 | 1.62436730732907E-12 |
|  | Mixed | Overexpression | 107 | 1.626032641866E-12 |
|  | Clear Cell | Overexpression | 2 | 9.727700E-02 |
|  | LBC-Non Mucinous | Overexpression | 19 | 8.05570000000921E-05 |
|  | Solid Pattern Predominant | Overexpression | 5 | 1.350110E-02 |
|  | Acinar | Overexpression | 18 | 5.020700E-03 |
|  | LBC-Mucinous | Overexpression | 5 | 3.172700E-04 |
|  | Mucinous carcinoma | Overexpression | 10 | 8.130200E-02 |
|  | Papillary | Overexpression | 23 | 5.80049999999721E-05 |
|  | Mucinous | Overexpression | 2 | 1.904000E-02 |
|  | Micropapillary | Overexpression | 3 | 1.870420E-01 |
|  | Signet ring | Overexpression | 1 | NA |
| Nodal metastasis status | Normal | Underexpression | 59 | - |
|  | NO | Overexpression | 331 | 1.62447832963153E-12 |
|  | N1 | Overexpression | 96 | 1.62447832963153E-12 |
|  | N2 | Overexpression | 74 | 1.62914126633495E-12 |
|  | N3 | Overexpression | 2 | 3.380000E-01 |
| TP53 mutation status | Normal | Underexpression | 59 | - |
|  | TP53-Mutant | Overexpression | 233 | 1.62447832963153E-12 |
|  | TP53-NonMutant | Overexpression | 279 | <1E-12 |

**Supplementary Table S3**. Correlation between *CKS1B* and selected clinicopathological features of LUSC.

| ***CKS1B* expression based on** | **Features** | **Expression of mRNA** | **Number of samples** | **Statistical significance (p-value)** |
| --- | --- | --- | --- | --- |
| Individual cancer stages | Normal | Underexpression | 52 | - |
|  | Stage-1 | Overexpression | 243 | 1.62447832963153E-12 |
|  | Stage-2 | Overexpression | 157 | 1.62436730732907E-12 |
|  | Stage-3 | Overexpression | 85 | 1.62447832963153E-12 |
|  | Stage 4 | Overexpression | 7 | 1.800600E-03 |
| Patient’s race | Normal | Underexpression | 52 | - |
|  | Caucasian | Overexpression | 343 | 1.62447832963153E-12 |
|  | African American | Overexpression | 30 | 7.10199676845491E-11 |
|  | Asian | Overexpression | 9 | 2.575700E-03 |
| Patient’s gender | Normal | Underexpression | 52 | - |
|  | Male | Overexpression | 366 | 1.62458935193399E-12 |
|  | Female | Overexpression | 128 | <1E-12 |
| Patient’s age | Normal | Underexpression | 52 | - |
|  | 21-40 Yrs | Overexpression | 2 | 4.383400E-01 |
|  | 41-60 | Overexpression | 103 | <1E-12 |
|  | 61-80 | Overexpression | 361 | 1.62447832963153E-12 |
|  | 81-100 | Overexpression | 20 | 1.68090000030041E-07 |
| Patient’s smoking habit | Normal | Underexpression | 52 | - |
|  | Non smoker | Overexpression | 18 | 2.47979999999925E-05 |
|  | Smoker | Overexpression | 133 | 1.62447832963153E-12 |
|  | Reformed Smoker 1 | Overexpression | 83 | <1E-12 |
|  | Reformed Smoker 2 | Overexpression | 247 | <1E-12 |
| Tumor histology | Normal | Underexpression | 52 | - |
|  | NOS | Overexpression | 480 | <1E-12 |
|  | Basaloid | Overexpression | 15 | 1.09579999962861E-07 |
|  | Papillary | Overexpression | 5 | 5.782000E-02 |
| Nodal metastasis status | Normal | Underexpression | 52 | - |
|  | NO | Overexpression | 320 | <1E-12 |
|  | N1 | Overexpression | 131 | 1.62447832963153E-12 |
|  | N2 | Overexpression | 40 | 1.90980564696019E-12 |
|  | N3 | Overexpression | 5 | 6.275100E-03 |
| TP53 mutation status | Normal | Underexpression | 52 | - |
|  | TP53-Mutant | Overexpression | 369 | 1.62436730732907E-12 |
|  | TP53-NonMutant | Overexpression | 118 | 1.62458935193399E-12 |

**Supplementary Table S4.** *CKS1B* expression and survival correlation in LC patients.

| **Dataset** | **Endpoint** | **Array type** | **Cox p-value** | **Hazard Ratio (HR)** | **Figure** |
| --- | --- | --- | --- | --- | --- |
| jacob-00182-CANDF | Overall survival | HG-U133A | 0.023684 | 2.18 | 6 (A) |
| jacob-00182-MSK | Overall survival | HG-U133A | 0.009744 | 2.17 | 6 (B) |
| GSE13213 | Overall survival | G4112F | 0.008198 | 1.66 | 6 (C) |
| GSE13213 | Overall survival | G4112F | 0.001003 | 1.93 | 6 (D) |
| GSE13213 | Overall survival | G4112F | 0.005499 | 1.76 | 6 (E) |
| GSE31210 | Overall survival | HG-U133_Plus_2 | 0.040969 | 1.91 | 6 (F) |
| GSE31210 | Relapse Free Survival | HG-U133_Plus_2 | 0.000535 | 2.20 | 6 (G) |
| GSE8894 | Relapse Free Survival | HG-U133_Plus_2 | 0.035593 | 1.25 | 6 (H) |
